# *Wolbachia w*Stri Blocks Zika Virus Growth at Two Independent Stages of Viral Replication

**DOI:** 10.1128/mBio.00738-18

**Published:** 2018-05-22

**Authors:** M. J. Schultz, A. L. Tan, C. N. Gray, S. Isern, S. F. Michael, H. M. Frydman, J. H. Connor

**Affiliations:** a Department of Biology, Boston University, Boston, Massachusetts, USA; b Molecular Biology, Cell Biology & Biochemistry Graduate Program, Boston University, Boston, Massachusetts, USA; c National Emerging Infectious Diseases Laboratories, Boston University, Boston, Massachusetts, USA; d Department of Biological Sciences, Florida Gulf Coast University, Fort Myers, Florida, USA; e Department of Microbiology, Boston University School of Medicine, Boston, Massachusetts, USA; Columbia University College of Physicians and Surgeons

**Keywords:** *Wolbachia*, arbovirus, arthropod vectors, biocontrol, mechanisms of action

## Abstract

Mosquito-transmitted viruses are spread globally and present a great risk to human health. Among the many approaches investigated to limit the diseases caused by these viruses are attempts to make mosquitos resistant to virus infection. Coinfection of mosquitos with the bacterium Wolbachia pipientis from supergroup A is a recent strategy employed to reduce the capacity for major vectors in the *Aedes* mosquito genus to transmit viruses, including dengue virus (DENV), Chikungunya virus (CHIKV), and Zika virus (ZIKV). Recently, a supergroup B *Wolbachia w*Stri, isolated from Laodelphax striatellus, was shown to inhibit multiple lineages of ZIKV in Aedes albopictus cells. Here, we show that *w*Stri blocks the growth of positive-sense RNA viruses DENV, CHIKV, ZIKV, and yellow fever virus by greater than 99.9%. *w*Stri presence did not affect the growth of the negative-sense RNA viruses LaCrosse virus or vesicular stomatitis virus. Investigation of the stages of the ZIKV life cycle inhibited by *w*Stri identified two distinct blocks in viral replication. We found a reduction of ZIKV entry into *w*Stri-infected cells. This was partially rescued by the addition of a cholesterol-lipid supplement. Independent of entry, transfected viral genome was unable to replicate in *Wolbachia*-infected cells. RNA transfection and metabolic labeling studies suggested that this replication defect is at the level of RNA translation, where we saw a 66% reduction in mosquito protein synthesis in *w*Stri-infected cells. This study’s findings increase the potential for application of *w*Stri to block additional arboviruses and also identify specific blocks in viral infection caused by *Wolbachia* coinfection.

## IMPORTANCE

Dengue, Zika, and yellow fever viruses are mosquito-transmitted diseases that have spread throughout the world, causing millions of infections and thousands of deaths each year. Existing programs that seek to contain these diseases through elimination of the mosquito population have so far failed, making it crucial to explore new ways of limiting the spread of these viruses. Here, we show that introduction of an insect symbiont *Wolbachia w*Stri, into mosquito cells is highly effective at reducing yellow fever virus, dengue virus, Zika virus, and Chikungunya virus production. Reduction of virus replication was attributable to decreases in entry and a strong block of virus gene expression at the translational level. These findings expand the potential use of *Wolbachia w*Stri to block viruses and identify two separate steps for limiting virus replication in mosquitos that could be targeted via microbes or other means as an antiviral strategy.

## INTRODUCTION

Arbovirus transmission is sustained as new viruses emerge and expand into new territories annually. Dengue virus (DENV) has been transmitted annually in the Americas (1) and Asia (2) for more than 20 years. Although there has been an efficacious yellow fever virus (YFV) vaccine for over 75 years (3), YFV continues to spread in Brazil (4) and Africa (5), with a fatality rate up to 60%. Chikungunya virus (CHIKV) cases are annually detected throughout Asia and the Americas (6). In 2015, symptomatic Zika virus (ZIKV) cases were at an all-time high, with millions of people infected (7) after this virus was imported into Brazil from Asia in 2013-2014 (8). The relentless transmission of arboviruses demonstrates a need for novel control measures. While each of these viruses cause a different pathogenesis in humans, ZIKV (9), DENV (10), YFV (11), and CHIKV (12) are all transmitted by the same *Aedes* species of mosquito. Thus, targeting the ability of the mosquito vector to support virus replication offers a novel way to uniformly limit arbovirus disease.

Several strategies to either reduce mosquito populations or reduce the capacity of a mosquito to transmit viruses have been studied (13). Insecticides, which globally reduce mosquito populations, have been met with the challenge of broad insecticide resistance (14). Alternatively, the sterile insect technique has had some success. Sterile (15) or transgenic male mosquitos (with a lethal development gene) (16) are released to crash mosquito populations. These strategies require costly annual maintenance, prompting investigation of self-sustaining methods to target mosquito-borne virus transmission.

One self-sustaining way to limit the capacity of a mosquito to transmit viruses is through coinfection with Wolbachia pipientis, an intracellular bacterium which already infects up to 40% of all arthropods (17). Many divergent strains of *Wolbachia* have coevolved with their arthropod host (18, 19) and are classified into supergroups. *Wolbachia w*Mel (supergroup A) was isolated from Drosophila melanogaster and has been transfected into Aedes aegypti mosquitos. A. aegypti mosquitos do not naturally have a *Wolbachia* infection (20). To establish *Wolbachia* infection in A. aegypti cultures, *Wolbachia* was first adapted to cell culture (21). This mosquito-adapted strain was used to stably infect A. aegypti mosquitos (22), and population introgression was demonstrated (20). This transinfection of *w*Mel has been shown to block DENV (20), ZIKV (23, 24), and CHIKV (25). As a result, *w*Mel field trials are now implementing *Wolbachia*-mediated biocontrol (26, 27).

Additional studies have demonstrated that other *Wolbachia* strains from the genetically distinct clade supergroup B may improve upon current *w*Mel strategies and offer important tools for investigating the mechanism of *Wolbachia*-mediated virus suppression. A. aegypti mosquitos stably infected with *w*AlbB (28), a strain native to A. albopictus mosquitos, are reportedly less permissive to DENV growth (29). *w*AlbB has also been shown to repress DENV (30) and ZIKV (31) in A. albopictus cell lines. A more distant supergroup B strain from Laodelphax striatellus, *w*Stri, has also been shown to repress ZIKV in A. albopictus cells. C/*w*Stri cells are an A. albopictus (C710)-derived cell line that is stably infected with *Wolbachia w*Stri. They are a powerful *in vitro* system for investigating the repression of viruses following *Wolbachia* colonization (31) because of their robust 4-log repression and because virus growth can be fully restored in these cells in the absence of *Wolbachia* replication. An additional advantage of these cells is that they lack a functional RNA interference pathway (32), allowing us to study mechanisms of virus suppression that are independent of this pathway.

The mechanism by which *Wolbachia* blocks viral growth is hypothesized to be multifaceted. We and others have shown that *Wolbachia* blocks viral growth early in infection—at or preceding genome replication (31, 33, 34). Conflicting studies have suggested that priming of innate immunity by the Toll/IMD pathway (35, 36) or small interfering RNA pathway (34, 37, 38) may play a minor role. RNA degradation and RNA methylation have also been suggested to alter viral growth (33, 39, 40). Additional work has implicated lipid composition or cholesterol in DENV inhibition in *Wolbachia*-infected cells (41). We previously showed that cholesterol-lipid supplementation partially rescues ZIKV growth in mosquito cells (31). However, the exact block in viral infection and how cholesterol contributes to this restriction are unknown.

We hypothesized that *Wolbachia* blocks viral growth at multiple stages of viral infection, based on the broad range of positive-sense RNA virus families that *Wolbachia* is able to repress. We first show that *Wolbachia w*Stri blocks positive-sense RNA viruses in both the *Flaviviridae* and *Togaviridae* families but not negative-sense RNA viruses in the *Bunyaviridae* and *Rhabdoviridae* families, demonstrating that *Wolbachia* blocks viral growth by a virus-specific mechanism. We further show that *Wolbachia w*Stri blocks positive-sense RNA virus growth at two independent stages of viral growth, based on studies using viral labeling, imaging, and reporter constructs for ZIKV in C/*w*Stri cells. Cholesterol-lipid supplementation partially rescued viral entry in *Wolbachia*-infected cells but did not rescue viral replication independent of entry. Delineating which viruses are repressed by *Wolbachia* and which stages of viral infection are blocked will focus future efforts to understand the mechanism by which *Wolbachia* limits viral growth.

## RESULTS

### *Wolbachia w*Stri inhibits positive-sense but not negative-sense RNA viruses.

**FIG 1:**
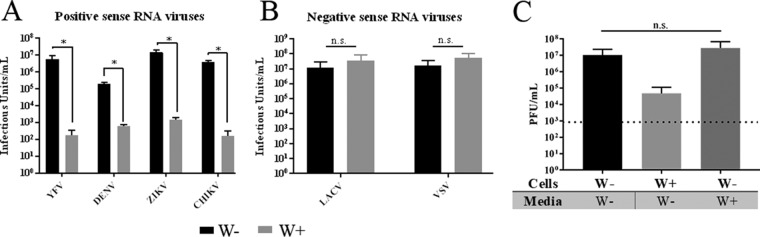
*w*Stri inhibits DENV and ZIKV but not LACV or VSV. *Wolbachia w*Stri-infected A. albopictus cells (W+) and uninfected *Wolbachia*-free control (W−) cells were infected with DENV-2, ZIKV, YFV 17 D, CHIKV, LACV, or VSV at an MOI of 10. After 3 days, virus supernatant was collected and infectious virus was assessed in a focus-forming assay (DENV and YFV) or plaque-forming assay (ZIKV, CHIKV, LACV, and VSV). Data shown are means and standard deviations of results from three independent experiments for each virus. (A) Positive-sense RNA virus genomes; (B) negative-sense RNA virus genomes. Statistical differences were determined with Student’s *t* test for each experiment. Where distribution of data was nonnormal due to no virus detected (YFV), the data were log transformed prior to the *t* test. *P* values were as follows: YFV, *P* = 0.003; DENV, *P* = 0.00004; ZIKV, *P* = 0.001; CHIKV, *P* = 0.003; LACV, *P* = 0.24; VSV, *P* = 0.26. (C) *Wolbachia w*Stri-infected A. albopictus cells (W+) and uninfected *Wolbachia*-free control (W−) cells were infected with ZIKV at an MOI of 10. Filtered medium from the spent W− culture or from the spent W+ culture was added to infected cells. Three days postinfection, cell supernatants were harvested and viral growth was assessed by plaque assay. *, *P* < 0.05.

Following experiments that showed that A. albopictus cells colonized with *w*Stri significantly reduced ZIKV replication compared with cells that were *w*Stri-free, we investigated the breadth of virus repression in *w*Stri-colonized cells. We chose a broad repertoire of viruses, including the positive-sense RNA viruses YFV, ZIKV, and DENV-2 (*Flaviviridae*), CHIKV (*Togaviridae*), and the negative-sense RNA arboviruses vesicular stomatitis virus (VSV) (*Rhabdoviridae*) and LaCrosse virus (LACV) (*Bunyaviridae*) for this investigation. *Wolbachia*-free cells (W−) and *Wolbachia*-containing cells (W+) were infected with each of these arboviruses at a multiplicity of infection (MOI) of 10, and virus accumulation in the medium was assessed at 3 days postinfection by plaque assay. Growth of YFV, DENV, and ZIKV (family *Flaviviridae*) was significantly reduced for all three viruses by *Wolbachia w*Stri (Fig. 1A) (*P* < 0.05, *t* test). YFV growth was repressed by approximately 4 logs, from a titer of 5.44 × 10^6^ focus-forming units (FFU)/ml in W− to only 1.67 × 10^2^ FFU/ml in W+ cells (99.99% reduction) (Fig. 1A). DENV-2 growth was similarly repressed in the presence of *w*Stri, showing growth of 1.99 × 10^5^ FFU/ml in W− cells but just 6.1 × 10^2^ FFU/ml in W+ cells (99.9% reduction) (Fig. 1A). Consistent with previous work (31), ZIKV growth was repressed approximately 4 logs in W+ cells (1.43 × 10^3^ PFU/ml) compared to W− cells (1.5 × 10^7^ PFU/ml) (Fig. 1A). Growth of CHIKV was significantly reduced (99.99%; *P* < 0.05, *t* test) in W+ cells (1.56 × 10^2^ PFU/ml) relative to that in W− cells (3.67 × 10^6^ PFU/ml) (Fig. 1A). These data are consistent with previous reports of supergroup A *Wolbachia* repression of positive-sense RNA viruses and demonstrate the broad-range antiviral phenotype for supergroup B *Wolbachia w*Stri.

In contrast to the repression of positive-sense RNA virus replication in *w*Stri-containing W− cells, negative-sense RNA virus growth was not inhibited in cells that contained *Wolbachia* (Fig. 1B). LACV growth in W+ cells (3.64 × 10^7^ PFU/ml) was slightly higher (not significantly) than in W− cells (1.18 × 10^7^ PFU/ml) (Fig. 1B). VSV growth was also unaffected (5.58 × 10^7^ PFU/ml in W+ compared to 1.69 ×10^7^ PFU/ml in W−) (Fig. 1B). These data showed that the restriction of positive-sense viruses was due to specific actions on positive-sense RNA virus replication and was not a general restriction of all virus replication.

### *Wolbachia*-conditioned medium does not repress ZIKV growth.

To better understand mechanisms of *Wolbachia*-mediated mosquito protection from positive-sense arboviruses, we sought to determine how and where virus replication is blocked in *Wolbachia*-infected cells. We focused on ZIKV PRVABC59 because of the significant interest in using *Wolbachia*-infected mosquitos as a ZIKV control strategy and the robust repression (>4 log) by *w*Stri. *Wolbachia*-mediated virus suppression has been suggested to be systemic throughout whole mosquitos (42). Thus, we first examined the hypothesis that an extracellular effector molecule secreted by *Wolbachia*-infected cells (43–45) was acting to repress viral growth. To test this hypothesis, we collected conditioned medium from W+ cells and conditioned medium from W− cells and assessed virus growth in W− cells in the presence and absence of medium from W+ cells. Virus growth in W− cells in the presence of W−-conditioned media was used as a control. W− cells treated with *W+*-conditioned medium grew to 2.80 × 10^7^ PFU/ml, which was comparable to the control W− cells treated with medium conditioned with W− cells (1.04 × 10^7^ PFU/ml) (Fig. 1C), suggesting that *Wolbachia* does not secrete an inhibitory factor outside the host cell to broadly repress viral growth. These data do not address if *Wolbachia* secretes an effector molecule into the host that alters the intracellular environment.

### *w*Stri inhibition of ZIKV is overcome at low *Wolbachia* infection frequencies.

**FIG 2:**
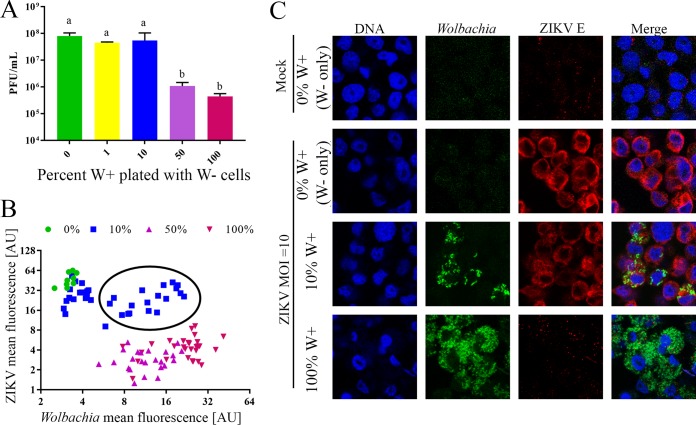
*w*Stri inhibition of ZIKV is overcome at low *Wolbachia* infection frequencies. *Wolbachia*-free (W−) cells were plated with varying quantities of *Wolbachia w*Stri-infected A. albopictus cells (W+) and incubated for 24 h to allow adherence. Cells were infected with ZIKV PRVABC59 at an MOI of 10 and incubated for 3 days. (A) Cell supernatant was collected and the viral titer was determined in a plaque-forming assay. The data shown are means and standard deviations of results from three independent experiments. Statistical differences in infectious virus were assessed by a one-way ANOVA followed by Tukey’s test for multiple comparisons. Conditions labeled “a” were statistically different from those labeled “b.” *, *P* < 0.05. (B and C) Cells were fixed and fluorescent *in situ* hybridization to the 16S ribosomal RNA gene was used to detect *Wolbachia* (green), and flavivirus envelope antibody (D11C) was used to detect ZIKV (red). Cells were counterstained with DAPI to indicate DNA (blue). *Wolbachia* and ZIKV concentrations per cell were calculated for each plating ratio by using ImageJ. Relative quantities of ZIKV and *Wolbachia* are shown in panel B. Each point is an individual cell. Representative images are shown in panel C. Parts of panel C were reproduced from reference 71, with permission.

We then investigated the possibility that cell-cell communication between W+ and W− cells might influence ZIKV growth. We cocultured W+ and W− cells at varying W+:W− cell ratios for 24 h. After cells adhered to the plate, they were infected with ZIKV at an MOI of 10. One hour post-viral absorption, the virus inoculum was removed and fresh medium was added to the cells. ZIKV growth was determined by plaque assay at 72 h postinfection. Results showed that there was a bimodal repression of ZIKV. When 10% or fewer W+ cells were plated with W− cells, ZIKV replicated to a titer of 4.47 × 10^7^ PFU/ml or higher, similar to growth in the control with W− only. When 50% or more of the plated cells were W+, ZIKV growth was strongly repressed (Fig. 2A) (one-way analysis of variance [ANOVA] and Tukey test, *P* < 0.05). These data suggest that infected cells do not produce an extracellular factor to systematically repress ZIKV replication and that *Wolbachia* infection frequency across a cell population is a determinant of antiviral phenotype.

From these data, it was unclear if *Wolbachia*-infected cells within a mixed *Wolbachia*-infected and *Wolbachia*-free cell population were replication sites for ZIKV or if they remained uninfected while W− cells produced virus. To understand if ZIKV was growing in *Wolbachia*-infected cells, we fixed cells from the described treatment, immunolabeled them for ZIKV, and probed them for *Wolbachia* by fluorescent *in situ* hybridization (FISH). Mean fluorescence of *Wolbachia* and ZIKV was determined in individual cells under each growth condition. ZIKV and *Wolbachia* concentration by mean fluorescence per cell were graphed against one another to determine if high ZIKV density occurs in the presence of *Wolbachia* (Fig. 2B). When 0% or 1% W+ cells were plated with W− cells, minimal *Wolbachia* infection and robust ZIKV growth were observed. In contrast, when 50% or 100% W+ cells were plated with W− cells, minimal ZIKV growth and high *Wolbachia* density were observed. Interestingly, when 10% W+ cells were plated with W− cells, both ZIKV and *Wolbachia* were observed in the same cells, perhaps due to the high titer of virus (Fig. 2B, black circle). These data suggest that *Wolbachia* infection frequency determines ZIKV growth *in vitro* but that, in cases of incomplete penetrance of *Wolbachia* infection in a cell population, repression is less effective and ZIKV can enter and/or replicate in cells.

### *Wolbachia w*Stri blocks ZIKV entry.

**FIG 3:**
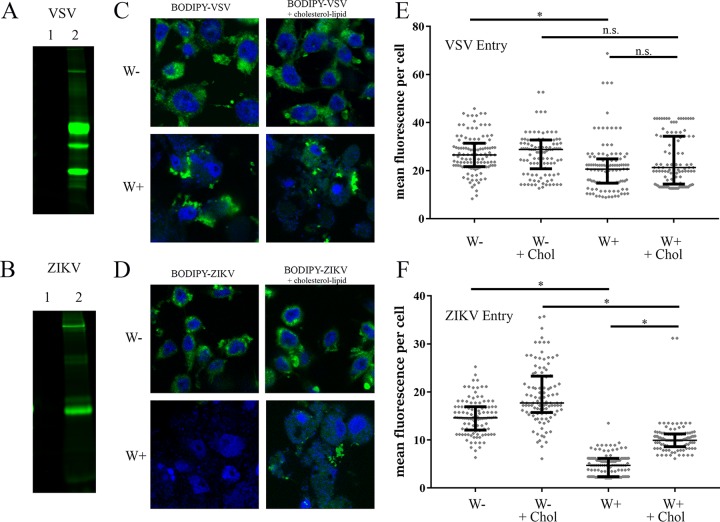
*Wolbachia w*Stri blocks ZIKV entry. (A and B) VSV (A) and ZIKV (B) were pelleted at high speed followed by resuspension in PBS buffer. Pelleted virus was labeled with a lysine conjugate, BODIPY 650/665. Virus labeling was visualized on a 10% SDS-PAGE gel and imaged at 700 nm to confirm BODIPY incorporation in glycoprotein and envelope protein, respectively. Lane 1, unlabeled virus; lane 2, labeled virus. (C to F) Labeled virus was incubated with W+ and W− cells at an MOI of 10 for 1 h at 28°C. After fixation, cells were mounted in Prolong Gold antifade medium with DAPI. (C and D) Representative images of BODIPY-virus incorporation. BODIPY-VSV (C) or BODIPY-ZIKV (D) are shown in green. DNA stained by DAPI is blue. (E and F) Individual cells were quantified for mean BODIPY intensity per cell for VSV (E) and ZIKV (F). Cell periphery was determined by differential interference contrast imaging. Reproducibility was confirmed through independent biological replicates. Statistically significant mean fluorescence was assessed by one-way ANOVA followed by Tukey’s test for multiple comparisons. *, *P* < 0.05.

Since a secreted effector molecule was not identified, we next investigated intracellular mechanisms of *Wolbachia*’s antiviral phenotype. Several reports have suggested that *Wolbachia* blocks virus replication at an early stage (31, 33, 34). We first tested whether *Wolbachia* blocked entry of virus. We labeled VSV and ZIKV with BODIPY 650/665 to create a fluorescently labeled virus particle that could be tracked in microscopy-based assays. When purified virus was analyzed by SDS-PAGE, no fluorescently labeled proteins were evident in the absence of BODIPY labeling (Fig. 3A and B, lane 1). Following BODIPY labeling (Fig. 3A and B, lane 2), fluorescent bands corresponding to VSV glycoprotein and ZIKV envelope were observed.

To test our hypothesis that *Wolbachia* blocks ZIKV entry, we incubated cells with ZIKV-BODIPY-labeled virus or VSV-BODIPY-labeled virus for 1 h at 28°C. Unattached virus was then removed and virus attachment and entry were determined by immunofluorescence. Resulting images showed that VSV entered both W− and W+ cells (Fig. 3C), yet ZIKV entry into W+ cells was severely impaired relative to entry into W− cells (Fig. 3D). Mean fluorescence intensity per cell was calculated to quantify viral entry for VSV (Fig. 3E) and ZIKV (Fig. 3F). The median of VSV-BODIPY mean fluorescent intensity in W− cells (26.5 arbitrary units [AU]) was decreased 22.2% compared to W+ cells (20.6 AU) (Table 1). Although this change was statistically significant, it was insufficient to alter VSV growth in W+ cells (Fig. 1A). ZIKV entry into W+ cells (4.7 AU) was reduced by 67.7% relative to that into W− cells (14.63 AU) (Fig. 3F; Table 2). These data show that ZIKV entry into *Wolbachia*-infected cells is reduced.

**TABLE 1:** VSV-BODIPY-labeled virus entry in W− and W+ cells

Cell type	Supplement	Fluorescence intensity (AU)	
		Median	% change from W−
W−		26.5	0.0
W−	Cholesterol	28.8	8.6
W+		20.6	−22.2
W+	Cholesterol	21.3	−19.7

**TABLE 2:** ZIKV BODIPY-labeled virus entry in W− and W+ cells

Cell type	Supplement	Fluorescence intensity (AU)	
		Median	% change from W−
W−		14.63	0.0
W−	Cholesterol	17.7	21.0
W+		4.728	−67.7
W+	Cholesterol	9.944	−32.0

Cholesterol has previously been implicated in flavivirus entry (46) and has been suggested to mediate *Wolbachia* repression of DENV (41). We previously showed that cholesterol-lipid supplementation partially rescues ZIKV growth in W+ cells (31). To assess if cholesterol-lipid addition altered ZIKV entry into W+ cells, we supplemented cells with an *in vitro* cholesterol-lipid reagent during viral absorption. Cholesterol-lipid supplementation had no significant effect on VSV entry (20.6 AU to 21.3 AU) (Fig. 3E; Table 2) but doubled ZIKV entry in W+ cells (4.7 AU to 9.9 AU) (Fig. 3F; Table 2). These results showed that cholesterol and lipids can increase ZIKV entry but that supplementation does not recover the entire replication block, suggesting that there are additional mechanisms.

### *Wolbachia* blocks viral replication downstream of ZIKV entry.

**FIG 4:**
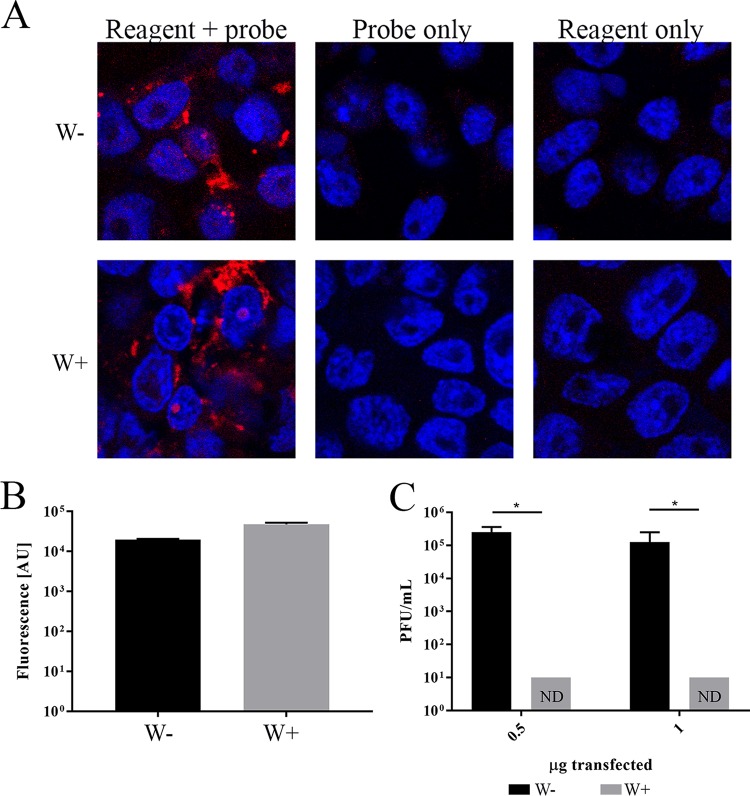
Independent of entry, *Wolbachia* blocks viral replication. (A and B) *Wolbachia w*Stri-infected cells (W+) and *Wolbachia*-free cells (W−) cells were transfected with 1 µg of ZIKV UTR probe with a AlexaFluor 547 conjugate (red) to assess transfection efficiency differences. (A) Cells were fixed and counterstained with DAPI (DNA; blue). Control cells received probe only without transfection reagent or reagent only without probe. (B) Fluorescence was detected by using a Tecan Spark plate reader at 547 nm. (C) ZIKV RNA was isolated by Trizol extraction, and 0.5 or 1 µg of viral RNA was transfected into W− or W+ cells. Four days posttransfection, supernatant was collected and virus production was quantified by plaque assay. The data shown are means and standard deviations of results from three independent experiments. Statistical significance was determined by using Student’s *t* test. *, *P* < 0.05.

To investigate *Wolbachia* blockade of viral replication downstream of viral entry, we tested whether transfected viral genomes were capable of producing infectious virus in W+ and W− cells. We initially assessed transfection efficiency in W+ and W− cells by using a fluorescently conjugated oligonucleotide. Transfection rates, determined by use of confocal imaging (Fig. 4A) and a plate reader (Fig. 4B), showed similar transfection efficiencies in W+ and W− cells. We then transfected 0.5 µg or 1 µg of purified viral RNA into cells and assessed the production of infectious virus 4 days posttransfection. W− cells produced greater than 1 × 10^5^ PFU/ml when either 0.5 or 1 µg of RNA was transfected (Fig. 4C). W+ cells failed to produce any detectable infectious virus when transfected with 0.5 or 1 µg of viral RNA. These data showed that a robust block in ZIKV growth occurs post-viral entry in *Wolbachia w*Stri-infected cells.

### *Wolbachia* blocks initiation of viral translation.

**FIG 5:**
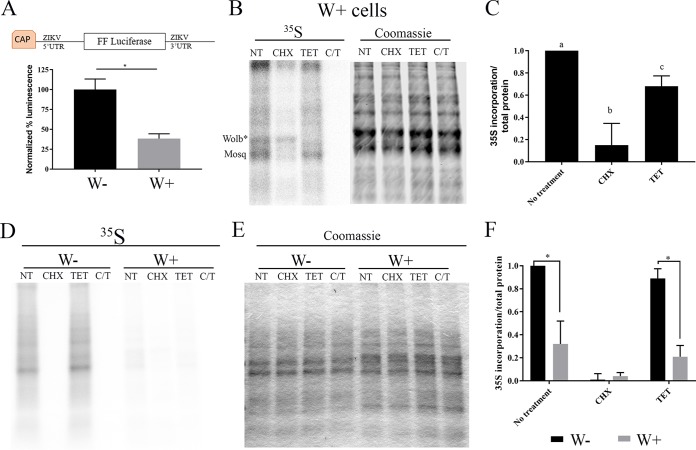
*Wolbachia* blocks initiation of viral translation. (A) ZIKV translation reporter construct diagram. The full 5′- and 3′-UTR of ZIKV flanking firefly luciferase were constructed in a pCDNA3 vector, amplified with T7 primers, *in vitro* transcribed, and capped. One microgram of translation reporter was transfected into W− and W+ cells. Twenty-four hours posttransfection, cells were lysed and luminescence was determined by using Nanolight reagent on a Tecan Spark plate reader. Data shown are the mean results of three independent experiments. (B) Quantitation of host translation in W+ cells demonstrated *Wolbachia* and mosquito translation by 30-min l-[^35^S]methionine pulse, 1 h before methionine starvation. (Left) l-[^35^S]methionine incorporation was measured by using a phosphorimager. (Right) Total protein was quantitated by Coomassie staining (right), read at 800 nm (infrared; Odyssey Licor). Lane 1, no treatment; lane 2, CHX at 10 µM; lane 3, TET, 22.5 µM; lane 4, CHX at 10 µM and TET at 22.5 µM. CHX and TET (C-T) were administered throughout starvation and a [^35^S]methionine pulse to block mosquito and *Wolbachia* translation. (C) Translation was quantified based on l-[^35^S]methionine incorporation relative to that for total protein. W− translation was compared to W+ translation based on l-[^35^S]methionine incorporation. Statistical differences were assessed by one-way ANOVA followed by Tukey’s test for multiple comparisons. (D to F) Each letter denotes statistical difference (*P* < 0.05) relative to total protein quantified by Coomassie staining (E) in panel F. For panels D and E: lanes 1 to 4, W−; lanes 5 to 8, W+; lanes 1 and 5, no treatment; lanes 2 and 6, CHX at 10 µM; lanes 3 and 7, TET at 22.5 µM; lanes 4 and 8, CHX at 10 µM and TET at 22.5 µM. Statistical differences were assessed by one-way ANOVA followed by Tukey’s test for multiple comparisons. *, *P* < 0.05 for three independent biological replicates performed for each experiment. Graphical representations show the means and standard deviations of independent biological replicates.

To determine whether a deficiency in viral protein production in W+ cells contributes to the blockade of ZIKV replication, we generated a reporter construct containing the 5′-untranslated region (UTR) and 3′-UTR of ZIKV flanking a luciferase reporter. One microgram of reporter was transfected into W− and W+ cells. W+ cells produced 68% less luminescence than W− cells, suggesting that ZIKV translation is significantly reduced in W+ cells (Fig. 5A) (*t* test, *P* < 0.05).

These results led us to examine whether this repression was due to a specific effect on ZIKV translation or whether *Wolbachia* infection resulted in a decrease in overall translation. We assessed the rates of translation in W+ and W− cells by pulse-labeling with [^35^S]methionine. Figure 5B shows the pattern of protein synthesis in W+ cells labeled with [^35^S]methionine for 30 min. Total protein measured by Coomassie staining (Fig. 5B, right) is shown to confirm equal loading. Under normal labeling conditions, a gray background punctuated with several prominently labeled bands was observed. Cycloheximide (CHX) treatment (lane 2) blocked most insect protein synthesis. Notable in CHX-treated cells was one prominent band that was translated at the same rate in the presence and absence of the eukaryotic protein synthesis inhibitor. This band disappeared when cells were treated with tetracycline, revealing that it is likely a *Wolbachia* protein (Wolb*) (Fig. 5B, left) that is produced through bacterial protein synthesis. Quantitation of these results indicated that *Wolbachia* protein translation represents approximately 1/3 of total protein translation (CHX), while 2/3 of total translation is mosquito derived (TET) in W+ cells (Fig. 5C).

There was a remarkable difference in the rate of protein synthesis observed when W− and W+ cells were labeled under similar conditions. As shown in Fig. 5D, there was significantly more total protein synthesis in W− cells than in W+ cells for a given amount of total protein present (Fig. 5E). As expected, CHX ablated all translation in W− cells (Fig. 5D, lane 1 compared to lane 2 or 4), and TET did not impair mosquito translation (Fig. 5D, lane 1 compared to lane 3). Quantification of the difference in protein synthesis (Fig. 5F) showed that when TET-treated W− cells were compared to TET-treated W+ cells, host translation was reduced approximately 66% in W+ cells. These data suggest that *Wolbachia w*Stri blocks viral translation by globally reducing host translation.

## DISCUSSION

The data presented here show that the *Wolbachia*-mediated inhibition of ZIKV replication in mosquito cells occurs by at least two independent mechanisms. We observed a significant reduction of ZIKV entry in cells infected with *w*Stri, which is likely to be an important cause of decreased ZIKV replication. Importantly, complementary experiments showed that if virion-mediated entry of ZIKV was bypassed, ZIKV replication was still blocked. This blockade is correlated with a reduction in the translation of a ZIKV minigenome reporter construct, indicating that replication is additionally blocked at the stage of protein synthesis. The finding that there are multiple mechanisms by which *Wolbachia* mediates viral repression is encouraging. If these multiple mechanisms of virus repression seen in cell culture are also active in an intact mosquito infected with *Wolbachia*, it is less likely that viruses will evolve resistance to this strategy of arbovirus control.

Our work also provides some insights into potential mechanisms for the different steps of virus restriction. We showed that cholesterol supplementation partially rescues the viral entry defect that we observed in W+ cells, which indicated that lower levels of cholesterol (known to occur in *Wolbachia*-infected cells [41]) limit ZIKV entry. While cholesterol-lipid supplementation was successful, it is important to note that we and others have been unable to rescue entry through the more common method of cholesterol-saturated methyl beta-cyclodextrin-mediated cholesterol supplementation (41). These results are consistent with our earlier findings (31) that showed cholesterol supplementation improved ZIKV replication.

Our data showing that ZIKV entry is affected in *w*Stri-containing cells in a cholesterol-dependent manner differ from those of a report that did not find a change in DENV entry into Aag2 cells infected with *w*Mel. Reasons for this discrepancy could stem from the differences in the virus used or *Wolbachia* strain used, but different assay approaches may play a role as well. Our results are based on direct imaging of labeled virus entry and include controls to affirm that known blockers of entry lead to decreases in signal in our assays. Thomas et al. used quantitative reverse transcription-PCR (40) without controls to define how known blockers of entry impact the results. It will be important to further assess these questions using similar assays in future experiments to improve our understanding of biological (mosquito/microbe) specificity versus technical limitations.

Our studies also demonstrated that there is a global decrease in protein synthesis activity in *Wolbachia*-infected cells. This global repression alone is a likely candidate for the lack of ZIKV protein production observed (31) and is consistent with other examinations of the effect of *Wolbachia* on insect cell homeostasis that have shown decreases in protein synthesis machinery (47). *Wolbachia* sequesters host amino acids, which may contribute somewhat to this decrease in mosquito translation (48). Proteomic interrogation of *Wolbachia*-infected cells has also indicated decreases in amino acid metabolism (47) and translation factors (49) following *Wolbachia* infection. The observed change in protein synthesis as a means of altering ZIKV replication is consistent with findings from other studies suggesting a block of virus replication early in viral replication (31, 34).

Global dysregulation of methylation patterns in *Wolbachia*-infected cells has previously been suggested to interfere with RNA virus replication. RNA methylation has been implicated in the translational control of host (50) and viral (51) protein production. *Wolbachia* disrupts global methylation patterns within a cell (52). Cytosine methyltransferases such as Dnmt2 have been shown to alter translation (53–55). Dnmt2 was downregulated in *Wolbachia*-infected mosquitos, limiting DENV replication (39), yet upregulated, limiting Sindbis virus, in *Drosophila* (33). Dnmt2 has also been shown to directly bind to Drosophila C virus RNA, a positive-sense RNA virus in *Drosophila* (56). These studies suggest dynamic control of host translation mediated by methylation of tRNAs. Additional studies are needed to delineate how global translation is downregulated in *Wolbachia*-infected cells and the specific effects on viral translation.

Our results are consistent with *Wolbachia* exerting part of its virus-repressive effect at the level of translation. This hypothesis is consistent with results from a study with a *Drosophila* cell line infected with *Wolbachia w*Mel; that study suggested that Semliki Forest virus replicons are inhibited early postentry at the stage of viral translation and/or replication, but it could not delineate between a block at one or both stages (34). Our results differ from those of another investigation of *Wolbachia*-virus interactions in *Drosophila* that suggested that *Wolbachia* repression of virus replication at RNA replication is caused by Dnmt2 (33). That study used a cyclic reporter to measure nonstructural versus structural open reading frame expression levels and suggested that translation was not hindered. In contrast to our study, this method is indirect and did not directly measure translation rates.

An additional factor that may contribute to *Wolbachia* restriction of flavivirus replication is the mosquito mRNA degradation system. A recent report showed that knockdown of XRN1 in *w*Mel-infected mosquito cells leads to a decrease in viral RNA elimination in these cells (40). This suggests that the degradation of viral RNA can contribute to the restriction of flavivirus replication and is consistent with our data showing changes at the level of protein synthesis. XRN1 is not functional on its own to degrade RNA that is capped. It requires the activity of the capping complex DCP2/DCP1, which recognizes and removes the 5′ cap (57). A reduction in recruitment of flavivirus RNA into the translation apparatus would leave the 5′ cap more vulnerable to decapping and increase its susceptibility to the XRN1 pathway.

While the global inhibition of translation may strongly affect ZIKV replication, it does not act as a universal block to viral growth. Our experiments demonstrate a different impact of *Wolbachia* infection on positive- and negative-strand RNA virus replication. In our experiments, *Wolbachia w*Stri infection only limited the replication of positive-sense RNA viruses. Our data are consistent with previous studies showing that *Wolbachia*-infected *Aedes* cells restrict the replication of ZIKV (23, 24, 31), DENV (26, 30, 37, 58, 59), and CHIKV (25, 60, 61). LACV and VSV have not been studied in *Aedes* mosquito cells in the presence and absence of *Wolbachia*, but the replication of the negative-sense RNA viruses is not impeded in *Wolbachia*-infected mosquitos. In Culex quinquefasciatus mosquitos (a nonnative vector of LACV), LACV was not restricted by *Wolbachia w*Pip (62). In Culex tarsalis mosquitos, a vector of the negative-sense Rift Valley fever virus, viral growth was also unaffected by *Wolbachia* (63). Thus, our data and recent literature expand a broad trend for *Wolbachia*-mediated viral repression of positive-sense but not negative-sense RNA viruses.

One way in which the protein synthesis block may have an effect on positive-sense RNA viruses but not negative-sense RNA viruses is the difference in replication strategies employed by the different viruses. Positive-sense RNA virus genomes are a singular infectious mRNA and must be translated by the host to generate a viral polymerase that will make additional mRNAs (64). Negative-sense RNA viruses bring with them a viral polymerase that generates mRNAs from the genome (64). The level of mRNA transcribed by this polymerase is regulated by the amount of viral protein produced. Under conditions where there is a general inhibition of protein synthesis, negative-sense RNA viruses would be predicted to overproduce mRNAs. This is done by extending the production of mRNA prior to translation (65), which raises the level of viral mRNA concentration until a sufficient amount of protein accumulates. Because positive-sense RNA viruses lack this mRNA production/compensation mechanism, a reduction in translation could lead to a much more severe decrease in protein synthesis.

Our studies also expand the understanding of the performance of the *w*Stri *Wolbachia* strain as a viral antagonist. Cells containing *w*Stri reduced the growth of all flaviviruses and alphaviruses (CHIKV) tested. Importantly, *w*Stri blocked YFV, which is not repressed by the supergroup A *Wolbachia* strain *w*Mel (66). These results suggest that *Wolbachia w*Stri has an ability to repress a wider variety of positive-sense RNA arboviruses and should be investigated *in vivo* for further applications. While *w*Mel is the strain currently employed in field trials (67), an understanding of *w*Stri alone or as a superinfection with *w*Mel *in vivo* may strengthen the approach of *Wolbachia*-mediated virus control.

Our study provides bases for multiple mechanisms of *Wolbachia*-mediated virus control and focuses future efforts to delineate molecular interactions which facilitate *Wolbachia*-induced viral repression. Additional studies investigating other flaviviruses as well as alphaviruses are needed to better understand the uniformity and divergence of how *Wolbachia* blocks specific viruses. Investigations of *w*Stri repression of viruses in A. aegypti cells and whole mosquitos are needed to advance the application of these studies. These data provide significant advancements in the understanding of how *Wolbachia* represses virus growth and offer new insights to the control of additional flaviviruses, such as YFV.

## MATERIALS AND METHODS

### Cell culture.

W+ cells (C/*w*Stri) were derived from W− cells infected with Wolbachia pipientis *w*Stri from Laodelphax striatellus (68), which was provided by Ann Fallon. W− and W+ cells were grown at 28°C with 5% CO_2_ and subcultured weekly at a 1:5 dilution in E-5 medium as previously described (31), with a modified increase of fetal bovine serum (FBS) concentration to 10%, termed E-10 medium. A. albopictus C6/36 cells were cultured in minimal essential medium (MEM) with 10% fetal bovine serum, 1× nonessential amino acids, and 2 mM glutamine and subcultured weekly at a 1:10 dilution. Macaca mulatta kidney LLC-MK2 (ATCC CCL-7) cells were cultured in Dulbecco’s modified Eagle medium (DMEM) with 10% fetal bovine serum and 2 mM glutamine at 37°C with 5% CO_2_. African green monkey (Cercopithecus aethiops) Vero E6 (ATCC CRL-158) cells were grown in MEM with 10% fetal bovine serum. African green monkey (Cercopithecus aethiops) kidney epithelial Vero cells (CCL-81; ATCC, Manassas, VA) were cultured in DMEM supplemented with 10% (vol/vol) fetal bovine serum, 2 mM Glutamax, 100 U ml^−1^ penicillin G, 100 µg ml^−1^ streptomycin, and 0.25 µg ml^−1^ amphotericin B at 37°C with 5% (vol/vol) CO_2_. LLC-MK2, Vero, and Vero E6 cells were subcultured biweekly at a 1:10 dilution.

### Viruses.

The following virus strains were used in this study: DENV-2 NGC, ZIKV PRVABC59, YFV 17 D, CHIKV 131/25, LACV H78, and VSV Indiana. DENV and ZIKV were propagated in C6/36 cells at an MOI of 0.01 for 6 days. YFV 17 D was recovered from a molecular clone obtained from Charles Rice at Rockefeller Institute. YFV 17 D was propagated in Vero cells at an MOI of 0.001 for 3 days. CHIKV was provided by Robert Tesh at the University of Texas at Galveston. Lyophilized CHIKV was reconstituted in serum-free DMEM and propagated in Vero cells for 2 days. LACV and VSV were grown in Vero E6 cells at a starting MOI of 0.01 and harvested when cytopathic effect showed greater than 50% lethality (approximately 24 to 36 h). Harvested supernatants were pelleted at 4,000 relative centrifugal force (RCF), filtered through a 0.2-µm polyethersulfone (PES) membrane, aliquoted, and stored at −80°C.

### Assessment of infectious virus.

Infectious units of ZIKV, CHIKV, LACV, and VSV were quantified by plaque assay. Briefly, Vero cells, or Vero E6 cells, were plated at ~90% confluence. Serial dilutions of virus were incubated with cells for 1 h followed by removal of the viral inoculum, and 1.4% Avicel in MEM with 10% FBS was added. Cells were incubated at 37°C with 5% CO_2_ for the following durations: ZIKV, 5 days; CHIKV, 2 days; LACV, 3 days; VSV, 1 day. After incubation, the overlay was removed by aspiration and cells were fixed with 4% formaldehyde for 1 h at room temperature. Crystal violet was incubated with cells for 10 min and cells were rinsed in water and counted.

DENV titers were determined in a focus-forming assay. Briefly, LLC-MK2 cells were incubated with serially diluted virus for 1 h. Virus was removed and cells were rinsed one time before adding 1.4% Avicel in MEM with 10% FBS. Plates were incubated for 72 h, followed by fixation in 10% formalin for 1 h at room temperature and then permeabilization with 70% ethanol for 30 min. Cells were stained with flavivirus anti-envelope protein antibody (D11C) (69) in phosphate-buffered saline (PBS) with 0.01% Tween 20 and 5% nonfat dry milk (NFDM), followed by goat anti-human antibody–horseradish peroxidase (HRP) conjugate. Foci were developed with 0.5 mg/ml diaminobenzidine in 25 mM Tris-HCl (pH 7.2). Foci were counted and results were graphed in GraphPad Prism.

The YFV 17D titer was determined in a focus-forming assay. Vero cells were seeded at a density of 5 × 10^5^ cells per well of a 12-well plate 24 h prior to infection. Serial dilutions of virus were incubated with cells for 1 h, followed by removal of the viral inoculum and addition of 1.4% Avicel in MEM with 10% FBS. Cells were incubated at 37°C with 5% CO_2_ for 48 h followed by fixation in Formalde-Fresh solution for 1 h at room temperature, and then they were permeabilized with 70% ethanol for 30 min. Cells were stained with flavivirus anti-envelope protein antibody (1.6D) (69) in PBS with 0.01% Tween 20 and 5% NFDM, followed by goat anti-human antibody–HRP conjugate. Foci were visualized by the addition of 3,3-diaminobenzidine tetrahydrochloride. Foci were counted and results were graphed in GraphPad Prism.

### High-MOI infections with positive- and negative-sense RNA viruses.

W+ and W− cells were seeded at 2 × 10^5^ cells per well in a 12-well plate and incubated at 28°C with 5% CO_2_ for 24 h. Cells were infected with one of the six viruses described above at an MOI of 10 in serum-free medium at 28°C with 5% CO_2_. After 1 h, virus inoculum was carefully removed and 1 ml of E-10 medium was added per well. Infections were incubated at 28°C with 5% CO_2_ for 72 h. Cell supernatant was collected and infectious virus was quantified as described above.

### Repressive capacity of *Wolbachia*-conditioned medium.

W+ and W− cells were seeded at 1 × 10^5^ cells in a total volume of 5 ml per T-25 flask and incubated at 28°C with 5% CO_2_ for 5 days. Conditioned medium was collected and pelleted at 4,000 RCF to remove any cells and cell debris. W+ and W− cells were seeded at 2 × 10^5^ cells per well in 12-well dishes. Cells were infected at an MOI of 10 with ZIKV PRVABC59 in serum-free medium for 1 h at 28°C with 5% CO_2_. Post-virus absorption, 1 ml of conditioned medium was added to cells. Infected cells were incubated at 28°C with 5% CO_2_ for 3 days. Supernatant was harvested, and infectious virus production was assayed as described above.

### Determining the effect of *Wolbachia* infection frequency on ZIKV growth.

W+ and W− cells were seeded in varying ratios of W+:W− cells with a total of 3.5 × 10^5^ cells per well in a 24-well plate and incubated at 28°C with 5% CO_2_ for 24 h. Cells were infected with ZIKV PRVABC59 for 1 h at 28°C with 5% CO_2_ in serum-free medium. Virus inoculum was removed and 0.5 ml of E-10 medium was added per well. Cells were incubated for 72 h at 28°C with 5% CO_2_. Postincubation, cell supernatant was collected to determine the production of infectious virus, as described above, and cells were processed for immunofluorescence as described below.

Cells were fixed with 4% paraformaldehyde with 0.1% Triton and 0.1% Tween 20 for 1 h at room temperature. Cells were then incubated in 70% ethanol for 30 min to further permeabilize the cells. Primary antibody to detect ZIKV envelope protein (D11C) was added at 1 µg/ml in PBS with 0.1% Tween 20. Cells were incubated overnight at 4°C. After removing and rinsing primary antibody, cells were incubated for 30 min with 1:500 goat anti-human antibody–Alexa Fluor 568 conjugate (secondary antibody). Secondary antibody was removed, and cells were rinsed three times with PBS and fixed with 4% paraformaldehyde a second time to stabilize the antibody complex during fluorescent *in situ* hybridization.

Fluorescent *in situ* hybridization was performed as previously described (70) with minor modification. Cells were then incubated in hybridization buffer (50% formamide, 5× SSC [1× SSC is 0.15 M NaCl plus 0.015 M sodium citrate], 250 mg/liter salmon sperm DNA, 0.5× Denhardt’s solution, 20 mM Tris-HCl, 0.1% SDS) at 37°C for 1 h. Next, 7.5 pg/µl 16S rDNA probe (AlexaFluor 488–5′-ACATGCTCCACCGCTTGTGCGGGTCCCCGTCAATT-3′) was added in hybridization buffer for a minimum of 3 h at 37°C, and then the mixture was washed in buffer 1 (1× SSC, 20 mM Tris-HCl, and 0.1% SDS) followed by wash buffer 2 (0.5× SSC, 20 mM Tris-HCl, and 0.1% SDS) at 37°C for 15 min each. Cells were stained with Hoechst at 0.5 µg/ml in wash buffer 2 for 30 min at room temperature. Finally, cells were rinsed with wash buffer 2 twice and mounted in Prolong Gold for imaging on an Olympus FV100 fluoview confocal microscope.

### BODIPY labeling and entry assay.

VSV and ZIKV were grown in 5 T-150 flasks as described above. Supernatant was pelleted at 4,000 RCF to remove cell debris and filtered through a 0.2-µm PES filter. Virus was pelleted at 32,000 rpm in an SW32Ti rotor in a Beckman Coulter, Inc. Optima L-90K ultracentrifuge. Virus pellets were resuspended in 1 ml of 1× PBS with 10 µg/ml BODIPY 650/665.Virus-BODIPY was incubated with rocking overnight at 4°C. Virus was collected and dialyzed in an 8,000-Da molecular weight cutoff membrane to remove unbound probe for 3 days in 3 liters of PBS at 4°C. PBS was exchanged for fresh solution each day. After dialysis, virus was collected and aliquoted. Virus titer was determined through a plaque assay as described above. Virus labeling was confirmed on a 10% SDS-PAGE gel. BODIPY 650/665 incorporation was determined through imaging on a Licor Odyssey in the 700-nm channel.

W− and W+ cells were plated at 3 × 10^5^ cells per chamber on a ChamberSlide (catalog number 154526; LabTekII) and incubated at 28°C with 5% CO_2_ for 24 h. Cells were infected with VSV-BODIPY or ZIKV-BODIPY for 1 h at 28°C with 5% CO_2_ in either serum-free medium or serum-free medium with cholesterol-lipid supplement at a 2× concentration (31). Virus inoculum was removed and cells were rinsed 3 times with PBS. Cells were fixed in 4% paraformaldehyde for 1 h at room temperature, mounted in Prolong Gold, and stained with 4′,6-diamidino-2-phenylindole (DAPI). Virus absorption was measured based on the mean BODIPY fluorescence per cell and using ImageJ.

### Transfection efficiency.

An AlexaFluor 568-conjugated probe was adhered to the 5′ end of the partial ZIKV 5′-UTR sequence: 5′-CTACTCCGCGTTTTAGCATATTGACAATCCGGAATCCTCCGG-3′. W+ and W− cells were plated at 3.5 × 10^5^ cells per well in 0.5 ml volumes in a 24-well plate and incubated for 24 h at 28°C with 5% CO_2_. Per well, 1 µg of probe was diluted in 100 µl of Optimem with 1 µl of Mirus Bio mRNA boost and 1 µl of Mirus Bio mRNA TransIT reagent; we allowed 3 min for lipid complexes to assemble and added them to cells. Control transfection experiments without the DNA probe or transfection boost and reagent were conducted simultaneously. Cells were incubated at 28°C with 5% CO_2_ for 24 h. Supernatant was removed, and cells were either fixed for imaging analysis or rinsed three times with PBS for plate reader analysis. For imaging, cells were fixed in 4% paraformaldehyde for 1 h at room temperature. Cells were rinsed 1 time with PBS and mounted in Prolong Gold and stained with DAPI. For plate reader analysis, cells were lysed in NP-40 lysis buffer and 100 µl of cell lysate was transferred to a clear-bottom plate. A Tecan Spark plate reader was used to determine fluorescence in the 568-nm channel.

### Infectious RNA isolation and virus genome transfection.

To isolate infectious viral RNA, five 150-cm^2^ flasks of C6/36 cells were infected with ZIKV at an MOI of 0.01. Six days postinfection, supernatant was pelleted at 4,000 RCF to remove cell debris and filtered through a 0.2-µm PES filter. Virus was pelleted at 32,000 rpm in an SW32Ti rotor in a Beckman Coulter, Inc. Optima L-90K ultracentrifuge. Virus pellets were resuspended in 500 µl of TRIzol reagent (Thermo Fisher Scientific). TRIzol RNA extraction was carried out per the manufacturer’s protocol. Viral RNA was resuspended in 100 µl of RNase-free water and quantified via a Thermoscientific Nano Drop 2000 system. W+ and W− cells were plated at 3.5 × 10^5^ cells per well in 0.5 ml volumes in a 24-well plate and incubated for 24 h at 28°C with 5% CO_2_. Each well was transfected with 0.5 µg or 1 µg of infectious RNA diluted in 100 µl of Optimem with 1 µl of Mirus Bio mRNA boost and 1 µl of Mirus Bio mRNA TransIT reagent. Three minutes was allowed for lipid complexes to assemble before being added to cells. Cells were incubated for 4 days at 28°C with 5% CO_2_. Twenty-four hours posttransfection cell supernatant was replaced with fresh media. At the end of the 4 day incubation, supernatant was collected and infectious virus was assessed by plaque assay as described above. RNA isolation by a Qiagen RNeasy extraction did not yield infectious RNA when transfected.

### Translation reporter construction.

A gene block with the 5′- and 3′-UTRs of ZIKV flanking the region encoding firefly luciferase was purchased from Integrated DNA Technologies, Inc. The 5′-UTR and 3′-UTR of ZIKV was derived from ZIKV strain FSS13025 (GenBank accession number KU955593.1). The gene block was cloned into a TOPO cloning vector and amplified with the following primers: ZIKV 5′-UTR with T7, 5′-GGCTTAATACGACTCACTATTAGAGTTGTTGATCTGTGTGAATC-3′; ZIKV 3′-UTR reverse primer, 5′-TGGGAATTCGGAAACCATGGATTTC-3′. For the amplification step, the PCR mixture was subjected to 40 cycles of 95°C for 30 s, 60°C for 1 min, and 72°C for 3 min. DNA was quantified by using a ThermoFisher Scientific Nano Drop 2000 system. One microgram of DNA was *in vitro* transcribed and capped by using an mMessenger mMachine T7 kit (catalog number AM1344; ThermoFisher Scientific). Capped RNA was isolated by using a Qiagen RNeasy kit per the manufacturer’s recommendations. W+ and W− cells were plated at 3.5 × 10^5^ cells per well in a 0.5-ml volume in a 24-well plate and incubated for 24 h at 28°C with 5% CO_2_. Each well was transfected with 1 µg of RNA reporter construct diluted in 100 µl of Optimem with 1 µl of Mirus Bio mRNA boost and 1 µl of Mirus Bio mRNA TransIT reagent. Lipid complexes were allowed to assemble for 3min before being added to cells. Transfected cells were incubated at 28°C with 5% CO_2_ for 24 h. Cell supernatant was removed and cells were lysed in 100 µl of NanoLight firefly luciferase reagent. Luminescence was determined on a Tecan Spark plate reader.

### l-[^35^S]methionine pulse treatment.

W− and W+ cells were seeded at 5 × 10^5^ cells/well in a 12-well plate and incubated for 24 h at 28°C with 5% CO_2_. Cells were methionine starved in Grace’s insect medium (-Met) supplemented with 1× nonessential amino acids for 1 h. Twenty-five microcuries of EasyTag l-[^35^S]methionine was added per well in Grace’s insect medium (-Met) for 30 min. To isolate *Wolbachia* versus host translation, 22.5 µM TET, 10 µM CHX, or both (C/T) were added to cells throughout starvation and l-[^35^S]methionine pulse. After the l-[^35^S]methionine pulse, cell supernatant was removed and cells were lysed in 100µl NP-40 lysis buffer with protease inhibitor for 5 min. Lysate was pelleted at 10,000 RCF to remove nuclei, and supernatant was collected. Supernatant was diluted in SDS loading buffer and heated to 95°C for 5 min. Protein lysate was run through a 10% SDS-PAGE gel and stained with Coomassie R-250 for 1 h. The gel was destained overnight in water and then imaged to measure total protein on a Licor Odyssey system in the 700-nm channel. The gel was dried at 80°C for 3 h on a vacuum drier. The dried gel was exposed to a phosphorimager for 2.5 h, and radioactive decay was read on a Bio-Rad imager. Radioactive decay was measured based on the intensity per lane and normalized to total protein per lane measured by Coomassie staining. C/T treatment showing background was subtracted from each lane per experiment. Means and standard deviations of three independent experiments were calculated in GraphPad Prism. One-way ANOVA followed by Tukey’s test determined statistical differences across samples.
